# Clinical Significance of *TP53* Abnormalities in Newly Diagnosed Multiple Myeloma

**DOI:** 10.4274/tjh.galenos.2021.2021.0064

**Published:** 2021-12-07

**Authors:** Fang Ye, Tongtong Wang, Aijun Liu, Yanchen Li, Ningning Li, Huan Wang, Wenming Chen

**Affiliations:** 1 Chuiyangliu Hospital Affiliated to Tsinghua University, Department of Hematology, Beijing, China; 2 Capital Medical University Beijing Chaoyang Hospital, Department of Hematology, Beijing, China

**Keywords:** TP53, Multiple myeloma, Genomic abnormality

## Abstract

**Objective:**

This study aimed to identify the clinical significance of *TP53* and common cytogenetic abnormalities.

**Materials and Methods:**

A total of 114 patients with newly diagnosed multiple myeloma (MM) and *TP53* abnormalities were selected from two large patient cohorts of collaborating hospitals from 2010 to 2017. The characteristics and outcomes of these patients were analyzed. *TP53* and other common mutations in MM patients were quantified by fluorescence in situ hybridization. Kaplan-Meier curves and log-rank tests were applied for survival analysis. A Cox proportional hazard model for covariate analysis was used to determine the prognostic factors.

**Results:**

By extensive data analysis, we found that *TP53* amplification is a strong positive predictor for complete response (CR) to therapy and positively correlated with patient survival. The number of simultaneous genomic abnormalities with *TP53* mutation has a modest impact on patient survival. Among these mutations, 1q21 amplification is associated with decreased CR (odds ratio: 4.209) and *FGFR3* levels are positively correlated with progression-free and overall survival.

**Conclusion:**

*TP53* abnormalities at the diagnosis of MM are of great clinical significance in predicting patient response to therapy and survival. Furthermore, 1q21 and *FGFR3* mutations could potentially be used in combination with *TP53* status to better predict patient survival and guide the selection of high-risk patients to advance patient treatment strategies.

## Abstract (tr)

**Amaç:**

Bu çalışma, *TP53*’ün klinik önemini ve yaygın sitogenetik anormallikleri belirlemeyi amaçladı.

**Gereç ve Yöntemler:**

2010 ile 2017 yılları arasında işbirliği yapan hastanelerin iki büyük hasta grubundan yeni teşhis edilmiş multipl miyelom (MM) ve *TP53* anormallikleri olan toplam 114 hasta seçildi. Bu hastaların özellikleri ve sonuçları analiz edildi. MM hastalarında *TP53* ve diğer yaygın mutasyonlar, floresan in situ hibridizasyon ile ölçülmüştür. Hayatta kalma analizi için Kaplan-Meier eğrileri ve log-rank testleri uygulandı. Prognostik faktörleri belirlemek amacı ile ortak değişken analizi için bir Cox orantılı tehlike modeli kullanıldı.

**Bulgular:**

Kapsamlı veri analizi ile, *TP53* amplifikasyonunun tedaviye tam yanıt (CR) için güçlü bir pozitif öngörücü olduğunu ve hastanın sağkalımı ile pozitif korelasyon gösterdiğini bulduk. *TP53* mutasyonu ile eşzamanlı genomik anormalliklerin sayısı, hastanın sağkalımı üzerinde sınırlı bir etkiye sahiptir. Bu mutasyonlar arasında, 1q21 amplifikasyonu, azalmış CR (olasılık oranı: 4.209) ile ilişkilidir ve *FGFR3* seviyeleri, progresyonsuz ve genel sağkalım ile pozitif olarak ilişkilidir.

**Sonuç:**

MM tanısındaki *TP53* anormallikleri, hastanın tedaviye yanıtını ve sağkalımı öngörmede büyük klinik öneme sahiptir. Ayrıca, 1q21 ve *FGFR3* mutasyonları, hasta sağkalımını daha iyi tahmin etmek ve hasta tedavi stratejilerini geliştirmek için yüksek riskli hastaların seçimine rehberlik etmek amacı ile potansiyel olarak *TP53* durumu ile kombine halde kullanılabilir.

## Introduction

Multiple myeloma (MM) is a hematologic malignancy caused by the proliferation of plasma cells in the bone marrow. It accounts for approximately 10% of all hematologic malignancies and 1% of all cancers [1,2]. The tumor plasma cells infiltrate bone marrow and other organs, which leads to lethal immune deficiency and organ damage [3,4,5]. Worldwide studies indicate that the incidence of MM has increased by 126% globally and the 5-year survival rate is only about 50% [6].

One major factor that contributes to the low survival rate is that MM is a highly heterogeneous disease, characterized by numerous genetic alterations [7]. Chromosome gains and losses, immunglobulin H translocations, and mutations of specific genes are often found in MM patients [7,8]. Genetic alterations are categorized as primary or secondary changes based on when the changes are observed during disease progression [9,10]. Cytogenetic abnormalities play a very important role in the survival of MM patients. For example, as determined by fluorescence in situ hybridization (FISH) detection, gain (1)(q21), del(17)(p13), and t(4;14)(p16;q32) in MM patients are correlated with shorter overall survival (OS) [11,12]. The fact that the type and quantity of genomic abnormalities are directly linked to MM patients’ survival time and response to treatment suggests that an investigation of the role of mutations in predicting patient response and survival is of great clinical significance in MM patient management [13].

Mapped to the position of chromosome 17p13, the *TP53* gene encodes the p53 protein and regulates the cell cycle. Since the discovery of the p53 protein, its role in cancer has been intensively investigated. p53 is an important tumor suppressor due to its critical role in inducing cell cycle arrest and apoptosis in response to cellular stress signals [14].

In MM patients, the major abnormalities of the *TP53* gene are mutation and deletion (due to deletion of the 17p13 region). These abnormalities of the *TP53* gene rarely occur at diagnosis; they increase in late-stage patients, suggesting the essential role of the *TP53* gene in disease progression [15,16]. Many clinical reports have shown a strong association between a loss of *TP53* and poor prognosis in MM patients [16,17,18,19]. However, due to the heterogeneity of MM and the limited number of cases, the function of *TP53* at diagnosis as a biomarker in different backgrounds of the major molecular cytogenetic abnormalities of MM is not well studied. Here, we provide an intensive retrospective analysis of a large cohort of newly diagnosed MM patients to identify the clinical significance of *TP53 *and common cytogenetic abnormalities. We compare *TP53* loss and amplification together with common genes dysregulated in MM patients, including chromosome 1q21 amplification, translocation of 4p16.3 (fibroblast growth factor receptor 3, *FGFR3*), and translocation of 16q23 (*MAF*) to chromosome 14q32. Investigation of the risk factors of MM relapse/progression will bring insight into the development of adaptive methods for better treatment of MM patients.

## Materials and Methods

### Patients

A total of 1046 newly diagnosed MM patients were enrolled from Beijing Chao-Yang Hospital, the Multiple Myeloma Research Center of Beijing, and Chuiyangliu Hospital Affiliated to Tsinghua University from January 2010 to December 2017. FISH was used to characterize the genetic abnormalities [*TP53*, 1q21, 14q32/11q13 (*CCND1* (cyclin D1 gene)], 14q32/4p16.3 (*FGFR3*), 14q32/16q23 (*MAF*) of these patients and diagnostic criteria were based on those of the International Myeloma Working Group [20]. Detailed criteria for FISH positivity are provided in Table 1. Basic patient information including age, gender, habits, baseline health, and comorbid diseases and clinical parameters including OS and chemotherapy response were recorded. The patient selection criterion was a primary diagnosis with *TP53* abnormality. Patients were excluded if they had refractory/relapsed MM. The study was approved by the ethics committee of our hospital. All patients gave written informed consent.

### FISH

FISH was performed on interphase cells. CD138-expressing plasma cells were purified and then FISH was performed as previously described [21] using probes purchased from Beijing Hightrust Diagnostic Company Limited. Targets detected by FISH and thresholds are included in Table 1. At least 200 plasma cells were scored to determine the prevalence of each genetic abnormality.

### Statistical Analysis

The primary endpoint of this study was correlated with survival from the time of diagnosis. Progression-free survival (PFS) and OS were evaluated according to the international uniform response criteria [22]. PFS was calculated from the time of diagnosis to the date of death, progression, or last follow-up. OS was defined as the duration from the time of diagnosis to the date of death or last follow-up. Descriptive statistics such as mean, standard deviation, median, and range were used for continuous variables while frequency counts and percentages were used for categorical variables. An independent sample t-test was employed to evaluate the associations between genetic abnormalities and biological parameters. The chi-square test or two-sided Fisher exact test was performed to make comparisons of categorical variables among groups. The Kaplan-Meier method was employed to plot survival curves, with a log-rank test to assess the differences. A Cox proportional hazard model for covariate analysis was used to determine the prognostic factors for PFS. All statistical analyses were performed using SPSS 17.0 (SPSS Inc., Chicago, IL, USA). The results were considered significant at p<0.05.

## Results

The median follow-up time for the entire population of MM patients was 32 months (range: 1-192 months). Among the 1046 newly diagnosed MM cases, *TP53* abnormalities were found in 153 cases, and 114 of those 153 cases (64 male patients, 50 female patients) were followed and included in the analysis, with a mean age of 59.4±10.3 years (Table 2). Among those 114 patients, 23 cases were stage I, 27 cases were stage II, and 64 cases were stage III at the time of diagnosis based on the International Staging System (ISS) (Table 2). Due to the significant effect of extramedullary disease (EMD) on survival rate reduction [23], patients’ EMD statuses at diagnosis were recorded. Most patients (86.84%) had no EMD at diagnosis (Table 2). Other medical history (hypertension, diabetes, heart disease, etc.), lifestyle factors (smoking and alcohol consumption), and clinical characteristics (neutrophils, platelet count, hemoglobin level, creatinine level, etc.) that may affect or reflect disease progression are provided in Tables 2 and 3. Patients mainly received autologous hematopoietic cell transplantation and/or standard chemotherapies, including but not limited to bortezomib combined with dexamethasone (PD) or three-drug combinations of PD with liposomal doxorubicin or thalidomide (Table 2).

**Figure 1:**
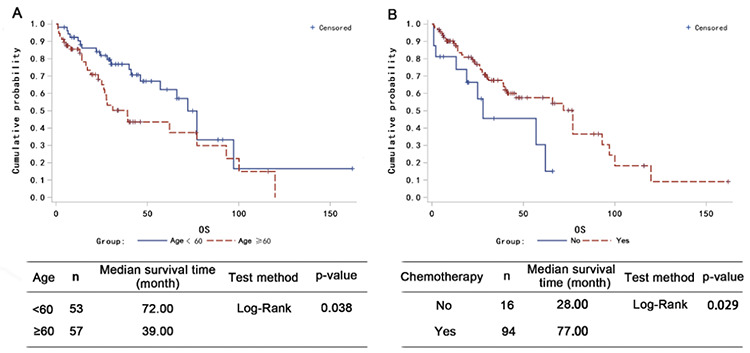
Factors that influence patient survival. Log-rank analysis of (A) age and (B) chemotherapy on patients’ overall survival. Patient numbers are indicated on the charts. Age groups are separated based on the mean age in our patient cohorts.

In our analysis, the OS of patients was mainly affected by age and chemotherapy. Younger age (<60 years old) correlated with increased OS rate compared to older patients (≥60 years old) (median survival: 72 months vs. 39 months, p=0.038) (Figure 1A). Chemotherapy increased the median survival time from 28 months to 77 months (p=0.029) (Figure 1B). However, the other major therapy received by our patients, autologous hematopoietic cell transplantation therapy, did not further improve patient survival rate (p=0.428; data not shown). Other factors including gender, ISS, Eastern Cooperative Oncology Group score, smoking, and alcohol consumption did not have a significant correlation with PFS or OS rates (data not shown).

**Figure 2:**
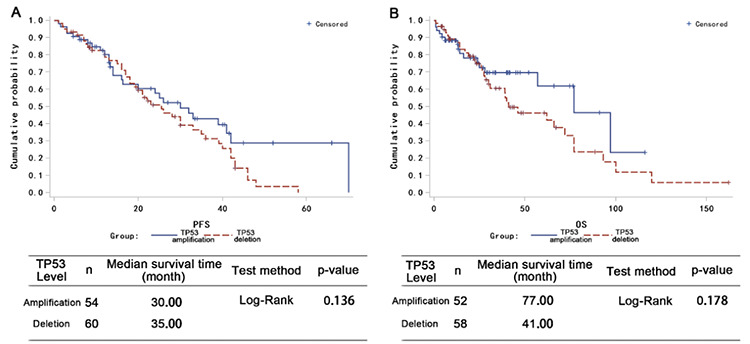
*TP53* level affects patient survival. Log-rank analysis of the effect of *TP53* amplification and deletion on (A) progression-free survival (PFS) and (B) overall survival (OS) of the patients.

**Figure 3:**
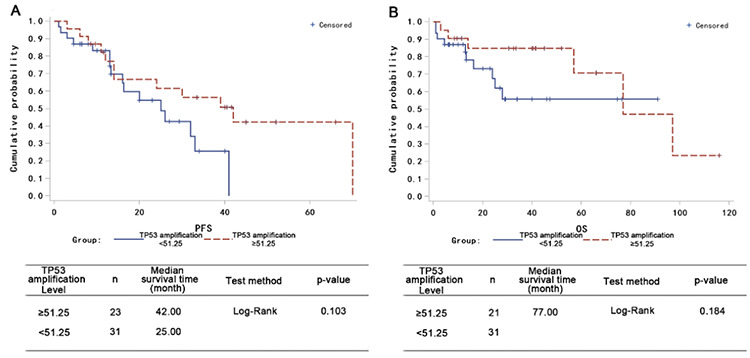
*TP53* amplification predicts better patient survival. Log-rank analysis of the effect of the level of *TP53* amplification on (A) progression-free survival (PFS) and (B) overall survival (OS) of the patients. The cutoff threshold of *TP53* amplification is based on receiver operating characteristic curve analysis.

Among the 114 patients with *TP53* abnormalities, 54 showed *TP53* amplification and 60 showed *TP53* deletion. Compared to patients with *TP53* deletion, those with *TP53* amplification had a higher probability of achieving complete response (Table 4; p=0.008) and had modest PFS and OS advantages (Figure 2A and 2B). When 3-year survival time was used as the cutoff in analysis, the patients who survived had a higher percentage of* TP53* amplification than patients who died (mean: 61.4% vs. 40.27%; p=0.034). The PFS and OS rates of patients with more than 51.25% *TP53* amplification (value calculated by receiver operating characteristic curve analysis; data not shown) trended more highly than those of patients with less *TP53* amplification (Figures 3A and 3B). Together, these data suggest that *TP53* amplification plays a positive role in patient survival.

The genes and chromosomes that are commonly dysregulated in MM patients were also tested in these 114 patients [chromosome 1q21 amplification, 4p16.3 (*FGFR3*), 16q23 (*MAF*), IgH translocations, abnormal chromosome counts] to show the potential effects of these common genetic dysregulations in the background of *TP53* abnormality. The genomic changes in these 114 patients are summarized in Table 5.

**Figure 4:**
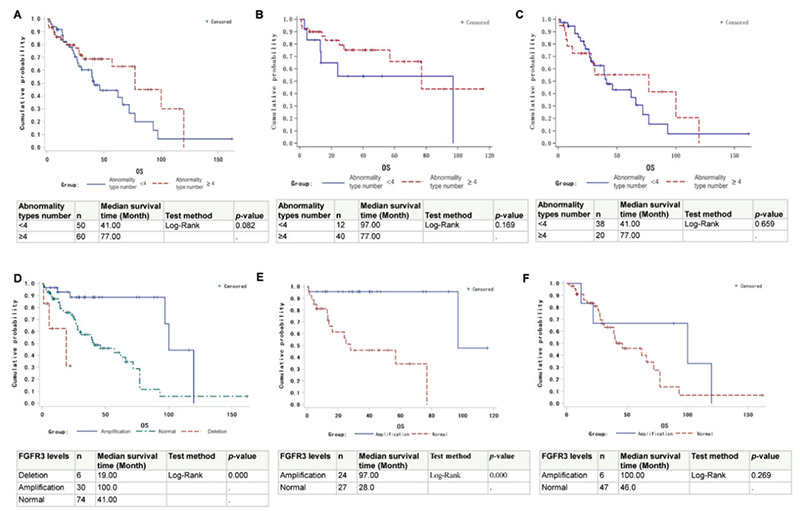
The ability of common mutations found in MM patients to predict patient survival among patients with *TP53* abnormalities. (A) Correlation between number of genetic abnormalities and patient OS. (B) Correlation between number of genetic abnormalities and patient OS in the background of *TP53* amplification. (C) Correlation between number of genetic abnormalities and patient OS in the background of *TP53* deletion. (D) *FGFR3* level in predicting median patient survival time. (E) *FGFR3* status in predicting median patient survival time in the background of *TP53* amplification. (F) *FGFR3* status in predicting median patient survival time in the background of *TP53* loss. MM: Multiple myeloma.

Overall, our data indicate that patients with four or more types of mutations in the list have PFS rates similar to those of patients with fewer than four types of mutation (data not shown). However, their OS rates trend more highly compared to patients with lower mutation burden (Figure 4A). When OS analysis was performed for patients separated by *TP53* status, though, no significant difference was found between patients with four or more types of mutations and patients with fewer than four types of mutations, potentially due to the low patient number in each group (Figures 4B and 4C). The effects of individual genetic abnormalities in the background of *TP53* abnormality on OS were also tested. In our patient cohorts with *TP53* abnormality, of the five genetic abnormalities (1q21, *FGFR3*, *MAF*, IgH translocations, and chromosome number changes), 1q21 amplification predicted the decreased probability of complete response (Table 4; odds ratio: 4.209), and the type of *FGFR3* mutation was critical in predicting patients’ PFS and OS. *FGFR3* amplification yielded a fivefold increase in median survival time compared to *FGFR3 *deletion (100 months vs. 19 months) and a twofold increase compared to patients with normal *FGFR3* (100 months vs. 41 months) (Figure 4D). We further analyzed median survival times for patients with *FGFR3* amplification and normal *FGFR3 *as separated by their *TP53 *statuses. Patients with *FGFR3* amplification still had significantly longer median survival time in the background of *TP53 *amplification (Figure 4E), but not in cases of *TP53 *loss (Figure 4F).

These data suggest that *TP53* status in combination with common mutations in MM could potentially be used to predict patient survival at the time of disease diagnosis.

## Discussion

*TP53* is a critical tumor suppressor and reported to correlate with MM disease progression. However, *TP53* mutation is a rare occurrence at diagnosis, being seen in only about 3% of newly diagnosed patients. The large patient cohorts in our hospitals provided an opportunity for us to study *TP53* mutation in early-stage MM patients, which brings insight into the clinical significance of *TP53* in newly diagnosed MM patients and also disease progression.

In 114 newly diagnosed MM patients with *TP53* abnormalities, we found that patient age and stage of the disease were the strongest predicting factors for patient PFS and OS, with older age and later stages indicative of worse prognosis, consistent with reports from other groups [24,25]. Patients’ lifestyles (smoking, etc.) and preexisting conditions (heart diseases, etc.) did not have strong effects on patient survival.

*TP53 *deletion is more commonly found in MM patients. In the present study we also reported a group of patients with *TP53 *amplification, which was associated with increased PFS and OS. The mechanism of *TP53* amplification is unknown, but it could potentially be caused by the compensating of non-functional p53 protein. Among the patients with *TP53* mutations, nearly half showed *TP53* amplification, and *TP53* amplification was a strong predictor for a complete response to therapy. Furthermore, the level of *TP53* amplification (≥51.25%) also showed a trend of positive correlation with patient survival rate. These data indicate that *TP53*, as a tumor suppressor, plays an important role in MM patient prognosis; patients with *TP53* deletion at an earlier stage and patients of older ages will potentially have a decreased chance of reaching complete response when treated with standard chemotherapy and autologous hematopoietic cell transplantation therapy. More advanced and intensive therapeutic strategies are potentially needed for these patients.

As common mutations in MM patients, 1q21 and *FGFR3 *levels were good predictors of patient’s therapy responses and OS in our cohorts. Copy number gain of chromosome 1q21 is among the most commonly reported genetic abnormalities in MM patients. The predictive role of 1q21 amplification in MM patients in terms of chemotherapy response and patient survival, however, is controversial. Studies have shown that 1q21 amplification strongly correlates with bortezomib resistance, but others showed no response prediction or survival benefit for patients with 1q21 amplification [26,27,28]. Our data indicate that in patients with *TP53* abnormalities, 1q21 amplification is a strong predictor for worse response to chemotherapy, suggesting that the study of 1q21’s role in the context of *TP53* mutation is of great clinical importance.

On the other hand, the t(4;14) translocation is associated with upregulation of *FGFR3* amplification, which has been shown to correlate with poor patient survival [29,30]. Interestingly, in contradiction with other studies, we found that in newly diagnosed MM patients with *TP53* mutation, *FGFR3 *levels had a strong positive correlation with patient PFS and OS. Patients with *FGFR3* amplification had a nearly twofold increase in median survival time compared to patients with normal *FGFR3* levels. These data suggest that *FGFR3* level is a critical prognosis indicator and a potential therapeutic target in MM patients with *TP53* mutation.

### Study Limitations

One limitation of our study is that the patient number is small, due to the fact that *TP53* mutation is rarely present at diagnosis. Data analysis for age or other mutation types is limited in the total population of patients with *TP53* mutation and separate analysis for each feature in *TP53* amplification and deletion could not be performed with statistical power. Another limitation of our study is that *TP53* mutation was tested at gene level. Whether the MM patients in our cohorts had functional p53 protein in their tumors or not is unknown, which may have introduced noise to our data analysis. Addressing the functional p53 protein levels in those patients in future work could potentially help to gain more statistical power in our analysis and a better understanding of the functional role of p53 in newly diagnosed MM patients.

## Conclusion

By extensive analysis of 114 newly diagnosed MM patients with *TP53* abnormalities, we observed a positive correlation between *TP53* amplification and MM patient survival. Further investigation of *TP53* and the common mutations in MM patients will contribute to the better design of biomarkers to predict MM patient therapy response and survival.

## Figures and Tables

**Table 1:**
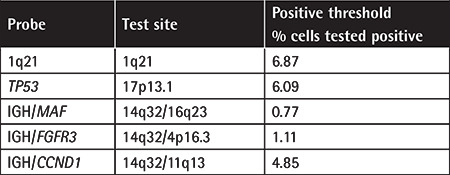
Summary of FISH positivity thresholds.

**Table 2:**
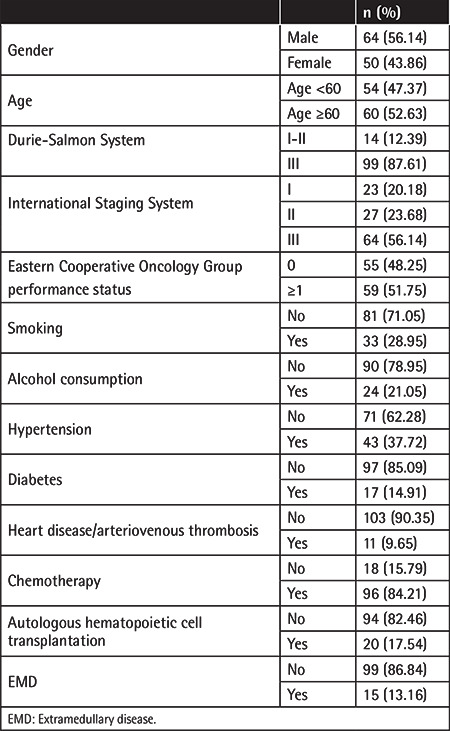
Summary of patients’ general information.

**Table 3:**
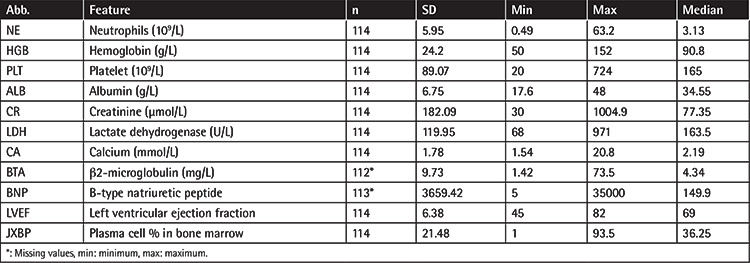
Summary of patients’ clinical features.

**Table 4:**
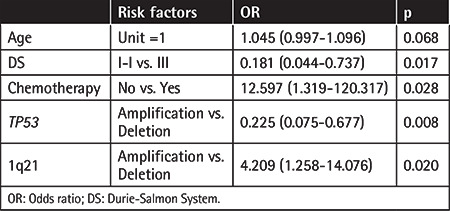
Risk factors involved in complete response to therapies.

**Table 5:**
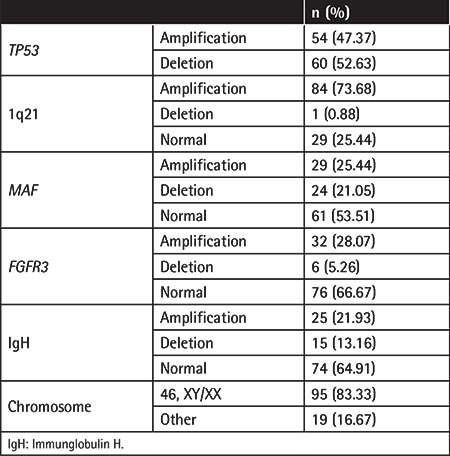
Summary of patients’ genetic abnormalities.
